# Polycystin-1 inhibits eIF2α phosphorylation and cell apoptosis through a PKR-eIF2α pathway

**DOI:** 10.1038/s41598-017-11526-0

**Published:** 2017-09-13

**Authors:** Yan Tang, Zuocheng Wang, JungWoo Yang, Wang Zheng, Di Chen, Guanqing Wu, Richard Sandford, Jingfeng Tang, Xing-Zhen Chen

**Affiliations:** 1 Department of Oncology and Haematology, The Second Hospital, Jilin University, 130041 Changchun, Jilin China; 2 Membrane Protein Disease Research Group, Department of Physiology, Faculty of Medicine and Dentistry, University of Alberta, T6G 2H7 Edmonton, AB Canada; 3 Division of Translational Cancer Research and Therapy, State Key Laboratory of Molecular Oncology, Cancer Hospital and Institute, Chinese Academy of Medical Sciences and Peking Union Medical College, 100021 Beijing, China; 4 Department of Medicine, Vanderbilt University, TN 37232 Nashville, Tennessee USA; 5 Academic Department of Medical Genetics, Addenbrooke’s Treatment Centre, Addenbrooke’s Hospital, CB2 0QQ Cambridge, Cambridge UK; 6 Institute of Biomedical and Pharmaceutical Sciences, Key Laboratory of Fermentation Engineering (Ministry of Education), Hubei Provincial Cooperative Innovation Center of Industrial Fermentation, Hubei Key Laboratory of Industrial Microbiology, Hubei University of Technology, 430068 Wuhan, Hubei China

## Abstract

Autosomal dominant polycystic kidney disease (ADPKD) is caused by mutations in *PKD1* or *PKD2* which encodes polycystin-1 (PC1) and polycystin-2, respectively. PC1 was previously shown to slow cell proliferation and inhibit apoptosis but the underlying mechanisms remain elusive or controversial. Here we showed in cultured mammalian cells and Pkd1 knockout mouse kidney epithelial cells that PC1 and its truncation mutant comprising the last five transmembrane segments and the intracellular C-terminus (PC1-5TMC) down-regulate the phosphorylation of protein kinase R (PKR) and its substrate eukaryotic translation initiation factor 2 alpha (eIF2α). PKR is known to be activated by interferons and dsRNAs, inhibits protein synthesis and induces apoptosis. By co-immunoprecipitation experiments we found that PC1 truncation mutants associate with PKR, or with PKR and its activator PACT. Further experiments showed that PC1 and PC1-5TMC reduce phosphorylation of eIF2α through inhibiting PKR phosphorylation. Our TUNEL experiments using tunicamycin, an apoptosis inducer, and GADD34, an inhibitor of eIF2α phosphorylation, demonstrated that PC1-5TMC inhibits apoptosis of HEK293T cells in a PKR-eIF2α-dependent manner, with concurrent up- and down-regulation of Bcl-2 and Bax, respectively, revealed by Western blotting. Involvement of PC1-regulated eIF2α phosphorylation and a PKR-eIF2α pathway in cell apoptosis may be an important part of the mechanism underlying ADPKD pathogenesis.

## Introduction

Autosomal dominant polycystic kidney disease (ADPKD) occurs with an incidence of ~1:1000 in all ethnic groups and develops as the result of mutations in the PKD1 (~70–85%) or PKD2 (~15–30%) gene, which encodes the protein product polycystin-1 (PC1) or polycystin-2 (PC2), respectively^1^. PC1 is a large 11-transmembrane protein containing an extracellular domain with Ig repeats and a short cytoplasmic domain that interacts with numerous signaling molecules^2, 3^. It is localized in cilia and at sites of cell-matrix and cell-cell interactions^4^. Animal models revealed that both loss- and gain-of-function of PC1 are cystogenic^5^. ADPKD is associated with dysregulated epithelial cell proliferation and apoptosis as well as elevated expression of oncogenes c-Myc and Bcl-2^6, 7^. Expression of PC1 in Madin-Darby canine kidney (MDCK) cells was reported to result in tubule formation and resistance to apoptosis^8^. PC1-inhibited apoptosis was linked to the phosphatidylinositol 3-kinase (PI3K)/Akt- and Gα_12_/Jun N-terminal kinases (JNKs)-dependent pathways^9, 10^. Interestingly, it was found that G protein α12 (Gα12) is necessary for the cystogenesis induced by dysregulated PC1 because lack of Gα12 in mice abolished PC1-dependent cyst formation^11^. Despite the tremendous progress made during the past two decades, the molecular mechanisms underlying ADPKD pathogenesis remain controversial.

Protein kinase R (PKR) was first identified in early 1990’s^12, 13^ but its existence in interferon (IFN)-treated vaccinia virus-infected L cells and its double-stranded RNA (dsRNA)-dependent kinase activity were known many years earlier^14, 15^. PKR is a 551-amino-acid (aa), 68-kDa ubiquitously expressed serine/threonine kinase composed of a catalytic C-terminus and a regulatory dsRNA-binding N-terminus containing two dsRNA-binding motifs^16^. It is also a pivotal antiviral protein and an essential component of the innate immunity that acts early in host defence prior to the onset of IFN counteraction and acquired immune responses^17^. Other than dsRNAs from cellular, viral or synthetic origins, PKR can be activated by Toll-like receptors, growth receptors and cytokines such as interleukin-1 and tumor necrosis factor α, and a variety of cellular stress inducers such as arsenite, thapsigargin and H_2_O_2_^18^. Further, the PKR-activating protein (PACT), which is activated by viral or non-viral stimuli, acts as a mediator that links a wide range of stress conditions to PKR activation^19^. Following binding of dsRNAs, PKR undergoes dimerization and auto-phosphorylation, and then phosphorylates its substrates, including eukaryotic translation initiation factor 2 alpha (eIF2α), protein phosphatase 2 A (PP2A) and IκB kinase (IKK). Through these substrates and downstream effectors PKR regulates translation, transcription and apoptosis^18, 20^. In addition to PKR, eIF2α is phosphorylated by three other kinases corresponding to different stress conditions: endoplasmic reticulum (ER) stress-activated protein kinase-like ER kinase (PERK), nutrient restriction-activated general control nonderepressible 2 (GCN2), and heme-regulated inhibitor (HRI)^21^.

Once activated by cellular stress, PKR inhibits proliferation and initiates apoptosis through phosphorylation of eIF2α to inhibit new protein synthesis, inhibition of B-cell lymphoma 2 (Bcl-2) function and activation of signaling pathways including nuclear factor (NF)-κB, p53, and signal transducer and activator of transcription 1^22, 23^. In addition, in response to stress conditions, increased phosphorylated eIF2α (P-eIF2α) up-regulates, both directly and through activating transcription factor 4 (ATF4), downstream effectors such as

homocysteine-induced ER protein (Herp) and C/EBP-homologous protein (CHOP)^21, 24, 25^. Apoptotic cell death is also ensued by ATF4-CHOP- mediated induction of several pro-apoptotic genes and by reduced synthesis of anti-apoptotic Bcl-2 proteins^26^. Likewise, the apoptosis induction by PKR involves phosphorylation of eIF2α, thereby regulating the expression of different genes such as pro-apoptotic Fas, Bcl-2-like protein-4 (Bax) and p53^27–29^. Interestingly, Bcl-2 was shown to block PKR-induced apoptosis^30, 31^. PKR-expressing cells contained elevated Bax and low levels of Bcl-2, while in cells expressing a catalytically inactive PKR variant, Bax was ablated and Bcl-2 was elevated^29^. Further, it was recently reported that the PKR/PP2A signaling axis is required for rapid and potent stress-induced apoptosis^32^.

To date, there has been no report about the relationship between PC1 and PKR involving apoptosis. In the present study, we employed cultured cell lines to investigate a PKR-dependent downstream pathway that is involved in mediating the effect of PC1 on apoptosis.

## Results

### Effects of PC1 on PKR and eIF2α phosphorylation in cultured cell lines and mouse kidneys

**Figure 1:**
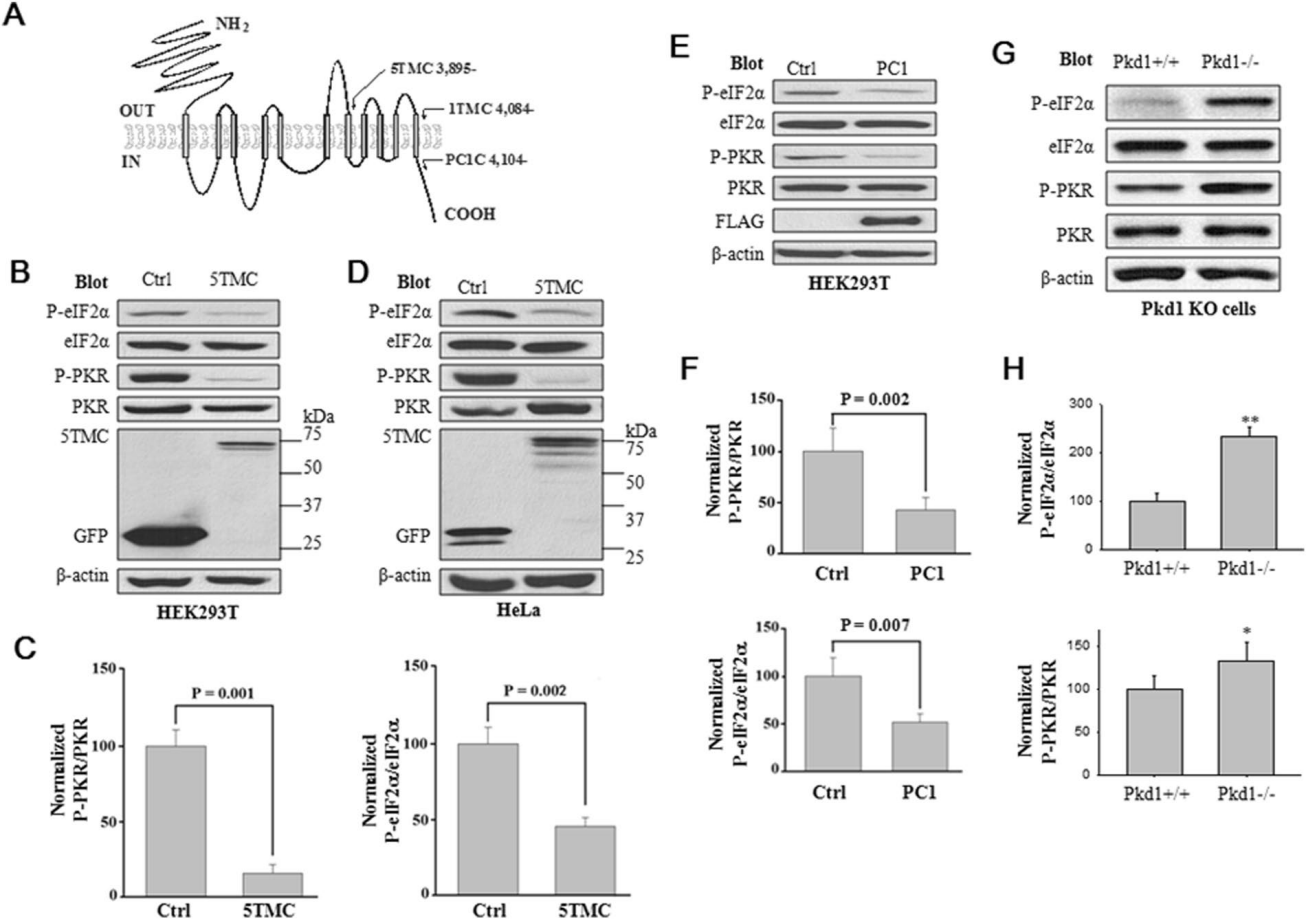
Down-regulation of P-eIF2α and P-PKR by PC1. (**A**) Schematic presentation of human PC1 membrane topology, showing 11 transmembrane domains, extracellular N- and intracellular C-termini. The numbers and positions of the starting amino acids for three truncation mutants are indicated. (**B**) Representative WB data. 60 µg of total proteins from HEK293T cells transfected with PC1-5TMC or GFP vector were loaded for immunoblotting. Blots were probed with the indicated antibodies. β-actin served as loading controls. Control (Ctrl), GFP vector; 5TMC, GFP-tagged PC1-5TMC. (**C**) Statistical data showing the relative activities (%) of eIF2α and PKR in HEK293T cells from panel B assessed by P-eIF2α/eIF2α and P-PKR/PKR, respectively. Shown are averaged P-eIF2α/eIF2α (N = 15, P = 0.002, paired t-test) and P-PKR/PKR (N = 10, P = 0.001, paired t-test). (**D**) Representative WB data using HeLa cells from similar experiments as in panel B. (**E**) Representative WB data using native HEK293T cells and those stably expressing mouse WT PC1. Cell were collected and loaded for immunoblotting by the indicated antibodies. Ctrl, native HEK293T cells; PC1, HEK293T cells stably expressing Flag-tagged full-length PC1. (**F**) Statistical data showing averaged ratios (%) of P-eIF2α/eIF2α (N = 9, P = 0.007, paired t-test) and P-PKR/PKR (N = 10, P = 0.002, paired t-test) from panel E. (**G**) Representative WB data showing the expression of P-eIF2α and P-PKR in mouse Pkd1 knockout mouse embryonic kidney epithelial cells. +/+, WT; −/−, Pkd1 homozygote. (**H**) Averaged and normalized ratios (%) of P-eIF2α/eIF2 and P-PKR/PKR from panel G are plotted. *p = 0.04, **p = 0.002 (N = 4, paired t-test).

We have previously reported that PC2 represses cell proliferation through promoting the P-eIF2α by kinase PERK^33^. Here we tested whether PC1 is involved in the regulation of eIF2α or its kinases. WB experiments using HEK293T cells revealed that expression of PC1-5TMC (aa 3,895–4,302), a truncation mutant of PC1 comprising of the last five transmembrane (TM) segments and the intracellular C-terminus (PC1C, aa 4,104-4,302), which contains the 20-amino acid (amino acids 4,134–4,153) fragment known to bind and activate G proteins^34^ (Fig. 1A), results in a decrease in the phosphorylated PKR (P-PKR) and P-eIF2α (Fig. 1B). In average, PC1-5TMC decreased the P-eIF2α/eIF2α ratio by 63.4% ± 7.7% (p = 0.001, N = 15) and P-PKR/PKR by 84.4% ± 9.1% (p = 0.001, N = 10) (Fig. 1C). Similar effects of PC1-5TMC were observed in HeLa cells (Fig. 1D). Furthermore, HEK cells stably expressing Flag-tagged mouse wild-type (WT) PC1 exhibited similar effects as PC1-5TMC on P-eIF2α and P-PKR (Fig. 1E and F). To determine whether PC1 has similar effects under more *in vivo* conditions, we also utilized primary epithelial cells prepared from Pkd1−/− knockout (KO) mouse embryonic kidneys for similar WB experiments. We found that the P-PKR/PKR and P-eIF2α/eIF2α ratios are increased in the Pkd1−/− cells as compared with control (Pkd1 +/+) cells similarly prepared from WT kidneys (Fig. 1G and H). These data together showed that PC1 down-regulates P-PKR and P-eIF2α in cultured cell lines and mouse kidneys.

### Interaction of PC1 with PKR and PACT

**Figure 2:**
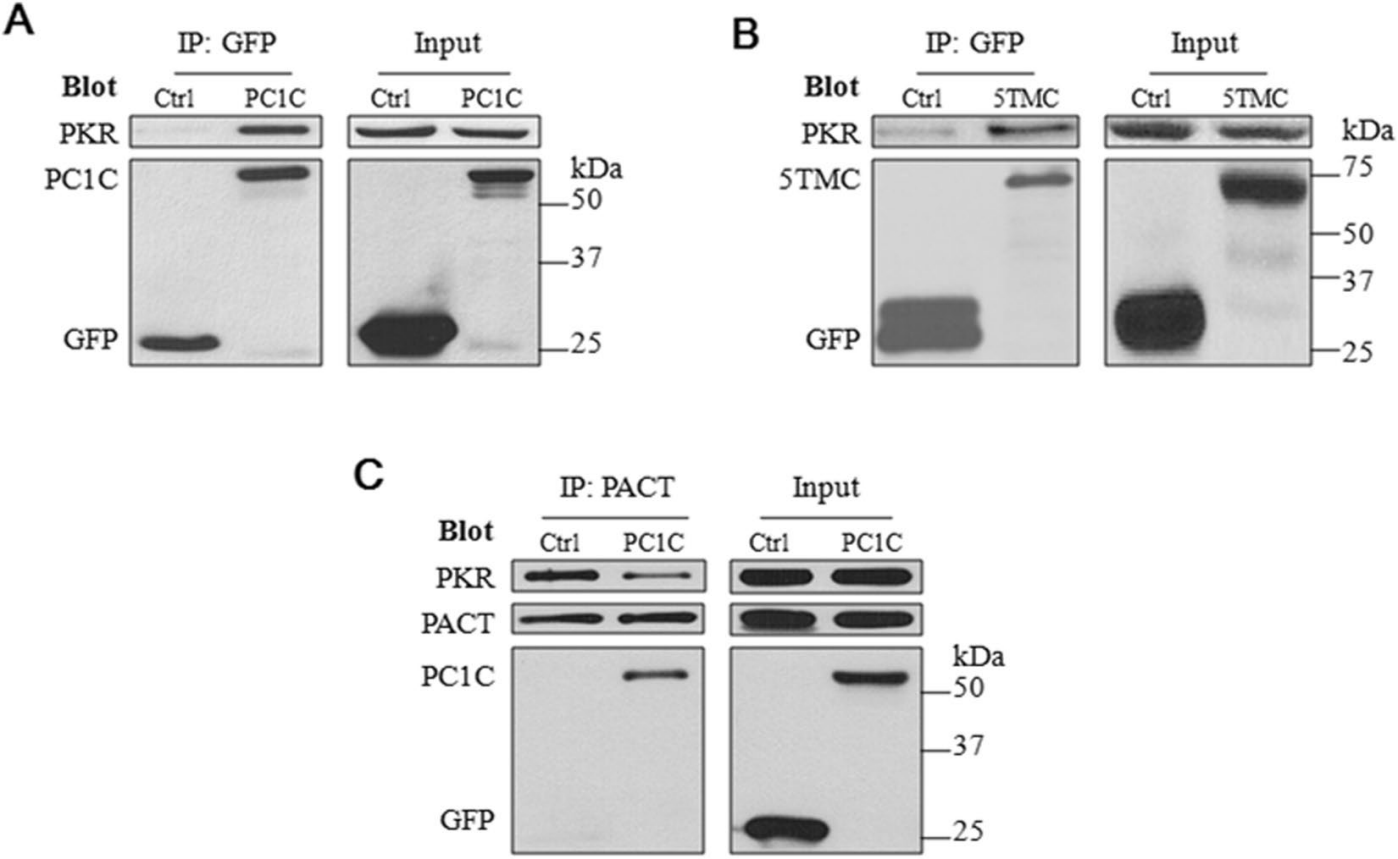
Interaction between PC1 truncation mutants, PKR and PACT by co-IP. (**A**) Representative data showing interaction between PC1C and PKR in HEK293T cells transiently transfected with pEGFP-PC1C or pEGFP. Total proteins from the transfected HEK293T cells were precipitated with anti-GFP (EU4), followed by SDS-PAGE and WB assays with anti-GFP (B-2). The blots were stripped and reprobed with the antibody against human PKR. Ctrl, GFP vector; PC1C, GFP-tagged PC1 C-terminus. (**B**) Representative data showing interaction between PC1-5TMC and PKR in HEK293T cells transiently transfected with pEGFP-PC1-5TMC or pEGFP. Experimental conditions were similar to those described in panel A. **(C)** Representative data showing interaction of PC1C with endogenous PKR and PACT in HEK293T cells. Total proteins from HEK293T cells were precipitated with anti-PACT antibody and detected with anti-GFP and anti-PKR antibodies.

We next examined whether there exists a physical interaction between PC1 and PKR. For this we performed co-immunoprecipitation (co-IP) experiments in HEK cells over-expressing PC1C or PC1-5TMC. We found that indeed PC1C and PC1-5TMC are able to precipitate PKR (Fig. 2A and B), suggesting that PC1 is in the same complex as PKR in HEK cells. Because the PKR activator PACT is known to bind with PKR^35^, we also immunoprecipitated with a PACT antibody and indeed found the PC1C, PKR and PACT signals in the precipitate, with reduced PKR band intensity in the presence of PC1C (Fig. 2C), suggesting that PC1C reduces the PKR-PACT interaction strength, possibly through competing with PACT for binding PKR.

### Down-regulation of P-PKR by the membrane-anchored PC1 C-terminus

**Figure 3:**
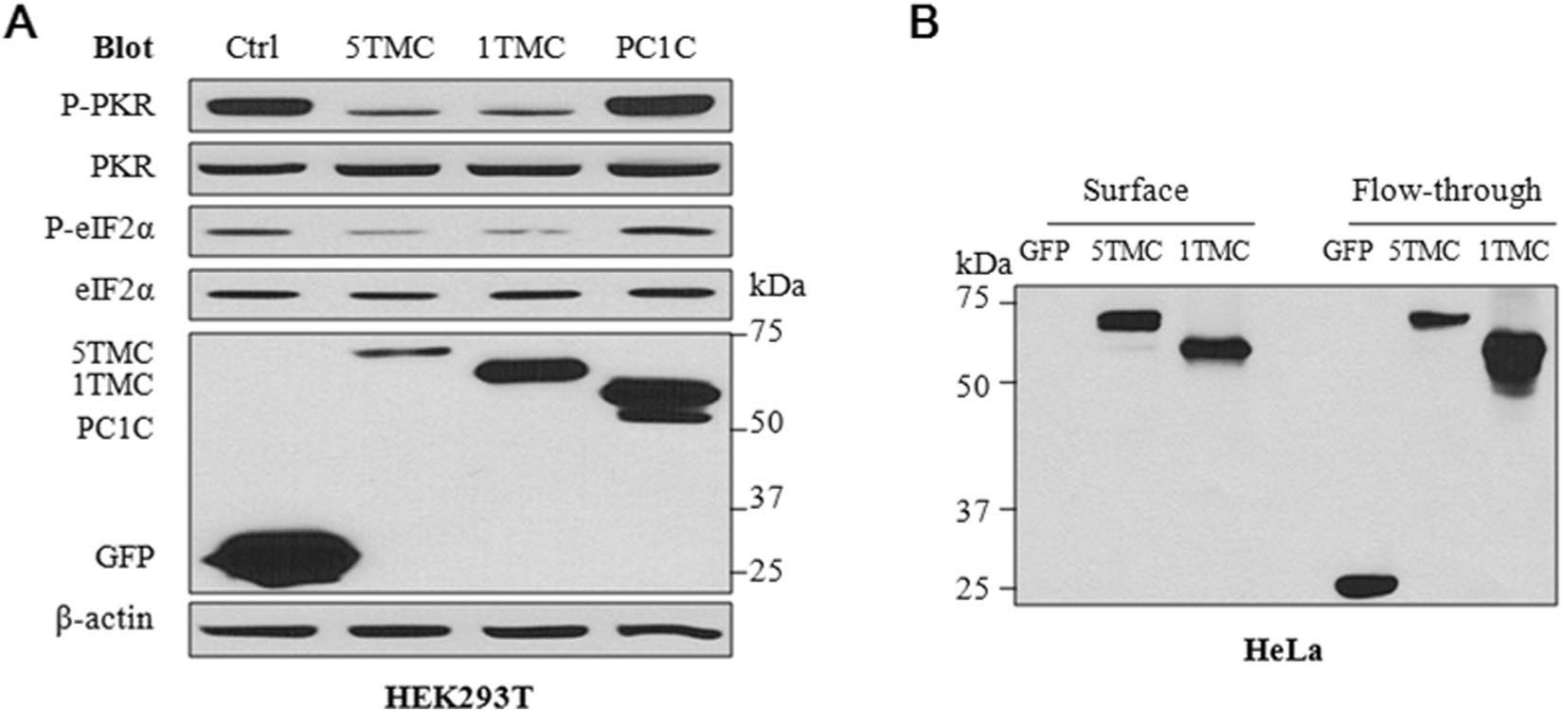
Effects of different PC1 domains on reducing the levels of P-PKR and P-eIF2α. (**A**) Representative data obtained using HEK293T cells transiently transfected with PC1-5TMC, PC1-1TMC, PC1C or GFP vector. Cell lysates were prepared for immunoblotting with the indicated antibodies. β-actin served as loading controls. 5TMC, GFP-tagged PC1-5TMC; 1TMC, GFP-PC1-1TMC; PC1C, GFP-tagged PC1 C-terminus. (**B**) Representative data obtained from surface biotinylation assays using HeLa cells transiently transfected with GFP-tagged PC1-5TMC or PC1-1TMC or GFP vector (control). Cells were incubated with 1 mg/ml Pierce EZ-Link^TM^ Sulfo-NHS-SS-Biotin. The biotinylated and flow-through intracellular proteins were separated using 100 µl of Pierce Avidin Agarose. An equal amount of biotinylated proteins and 20 µg of total and intracellular proteins were separated on 8% SDS-PAGE for immunoblotting.

To determine which part of PC1 is required for the reduction of the P-PKR level, we transfected human PC1-5TMC, PC1-1TMC and PC1C into HEK293T cells, and found by WB assays that while PC1-1TMC (aa 4084-4302) (Fig. 1A) has a similar inhibitory effect on P-PKR as PC1-5TMC, PC1C does show any significant effect (Fig. 3A). Using cell surface biotinylation assays we found that green fluorescent protein (GFP)-tagged PC1-1TMC and -5TMC, but not GFP alone, traffic to the plasma membrane (PM) of HeLa cells (Fig. 3B). These data together suggest that the PM-anchored PC1 C-terminus but not the PC1 C-terminus alone confers the down-regulation of the PKR activity.

### Dependence of the down-regulation of P-eIF2α by PC1 on the kinase activity of PKR

**Figure 4:**
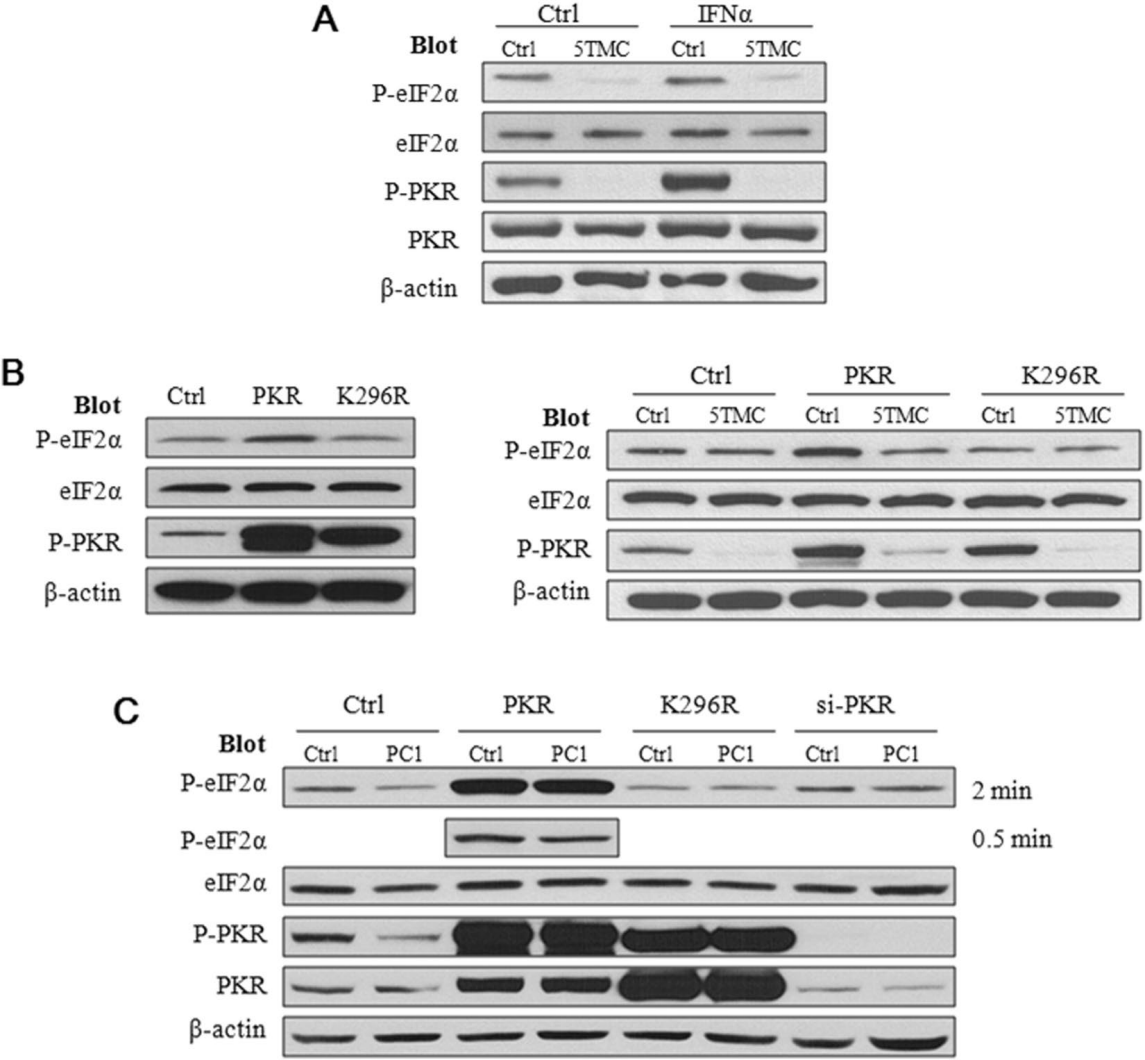
Dependence of the PC1-regulated eIF2α activity on P-PKR kinase activity. (**A**) Effect of IFNα. Representative data showing the effects of PC1-5TMC on the activity of PKR and eIF2α in the presence and absence of IFNα in HEK293T cells that were transfected with GFP-tagged PC1-5TMC or GFP vector and treated with IFNα at 1,000 U/ml for 24 hours. Blots were probed with the indicated antibodies. (**B**) Left panel: Effects of the PKR K296R mutation on the PKR and eIF2α activity. HEK293T cells transfected with WT PKR, PKR-K296R or GFP vector. Right panel: Roles of the PKR K296R mutation on the regulation of the PKR and eIF2α activity by PC1 truncation mutant. HEK293T cells transfected with PC1-5TMC or GFP vector were co-transfected with WT PKR or PKR-K296R. Blots were then probed with the indicated antibodies. Ctrl, GFP vector; PKR, WT PKR; K296R, PKR-K296R. (**C**) Roles of PKR expression and kinase activity on regulation of the PKR and eIF2α activity by PC1. Native HEK293T cells and those stably expressing WT PC1 were transiently transfected with WT PKR, PKR-K296R or PKR siRNA before blots were probed with the indicated antibodies. For P-eIF2α blots in the presence of PKR over-expression, bands obtained with a shorter exposure time (0.5 min, vs regular exposure of 2 min) was also shown to avoid band saturation.

We next examined whether PC1-mediated down-regulation of P-eIF2α involves the activity of the eIF2α kinase PKR, using IFN-α to activate PKR. We first found that PC1-5TMC abolishes the up-regulation of P-PKR and P-eIF2α by the inducer (Fig. 4A). We then transfected HEK cells with PKR and dominant negative PKR mutant K296R that retains the auto-phosphorylation ability but has no kinase activity^36^. As expected, while the PKR over-expression increased both the P-eIF2α and P-PKR level, over-expression of mutant K296R has no effect on eIF2α phosphorylation (Fig. 4B, left panel), confirming that mutant K296R has no kinase activity. Consistently, we found that PC1-5TMC inhibits the phosphorylation of both the WT and mutant PKR (Fig. 4B, right panel). Consequently, PC1-5TMC substantially reduced the P-eIF2α level in cells over-expressing PKR but had no significant effect on P-eIF2α in those over-expressing mutant K296R (Fig. 4B, right panel). We found that full-length PC1 and PC1-5TMC in general have similar effects on the P-eIF2α level (Fig. 4C). Taken together, our data suggest that PC1 inhibits PKR phosphorylation through which it down-regulates the eIF2α activity.

We next utilized PKR knockdown by siRNA to further document the down-regulation of P-eIF2α through inhibiting PKR phosphorylation. By using and comparing HEK293T cells with and without stable expression of Flag-tagged full-length PC1, we found that either PKR knockdown or mutant K296R over-expression abolishes the down-regulation of P-eIF2α by WT PC1 (Fig. 4C). These data are in general in support of our conclusion that PC1 represses eIF2α phosphorylation through down-regulating PKR kinase activity. We noticed that PKR knockdown has little effect on eIF2α phosphorylation (Fig. 4C), suggesting the possibility that the basal levels of P-eIF2α be mainly attributed to a protein phosphatase or an eIF2α kinase other than PKR.

### Dependence of PC1-inhibited apoptosis on a PKR-eIF2α pathway

Because PC1 and PKR were respectively known to inhibit and induce cell apoptosis^8, 9, 18, 37^, we wondered whether PC1 inhibits apoptosis through down-regulating the PKR and eIF2α activities. For this purpose we carried out TUNEL assays to determine apoptosis of HEK293T cells after transfection of PC1-5TMC and Western blot (WB) assays to evaluate the P-eIF2α/eIF2α or P-PKR/PKR ratios, in the presence and absence of tunicamycin (Tm) as an apoptosis inducer^38^.

**Figure 5:**
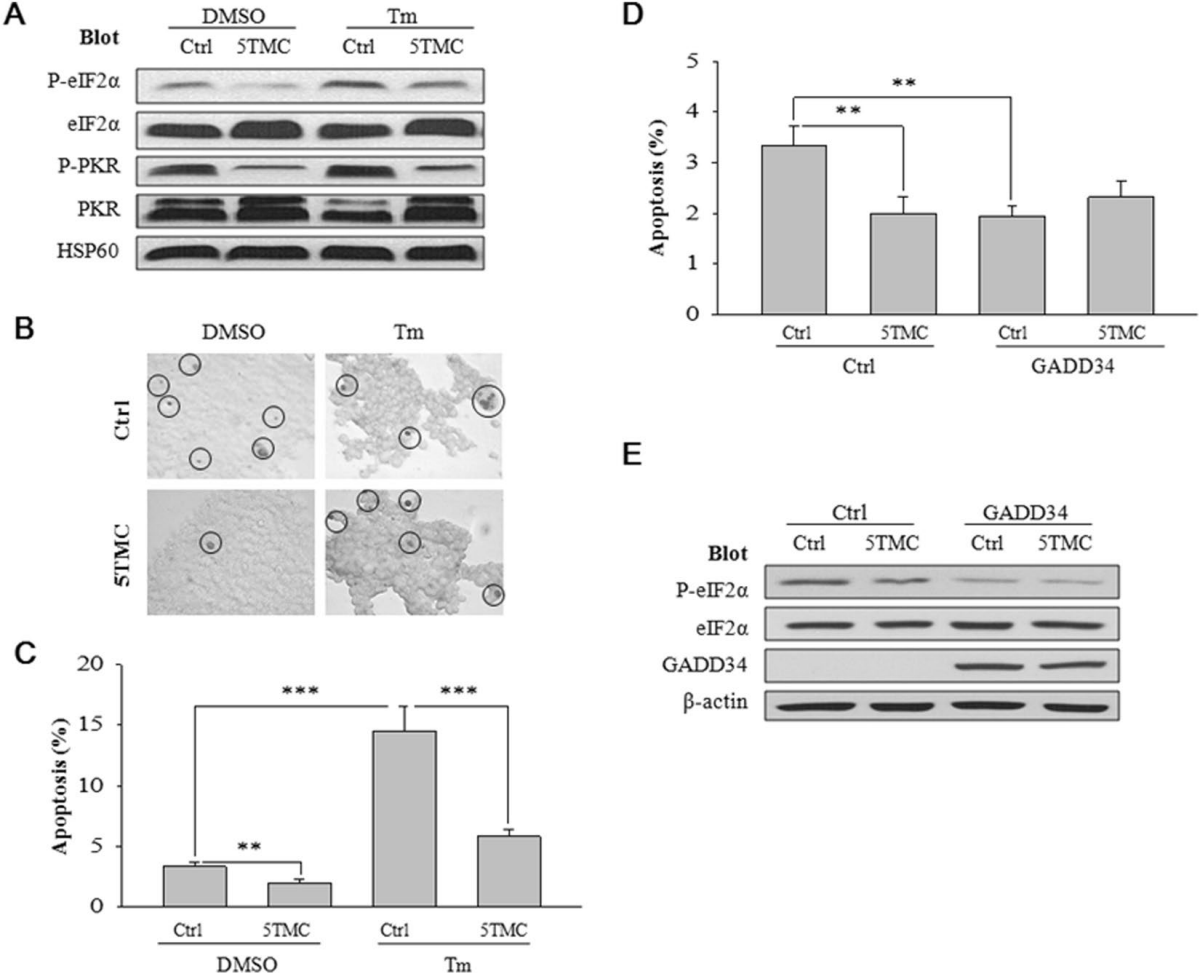
Dependence of PC1-inhibited apoptosis on P-eIF2α/P-PKR. (**A**) Effects of PC1 and Tm treatment on P-eIF2α and P-PKR. HEK293T cells were transfected with GFP-tagged PC1-5TMC (5TMC) or GFP (Ctrl) and treated with Tm (2.5 µM in 5% dimethyl sulphoxide (DMSO)) or DMSO (5%, as control) at 24 hours post-transfection. After 24 hours, cell lysates were loaded for immunoblotting. HSP60 served as a loading control. (**B**) Photomicrographs of HEK293T cells used for TUNEL assays (magnification × 200). HEK293T cells were transfected with PC1-5TMC or GFP vector before being treated with DMSO or Tm and performed as in Fig. 5A. TUNEL-positive cells were indicated by circles. (**C**) Statistical data from TUNEL assays similarly performed as in panel B and averaged to compare apoptotic levels in HEK293T cells treated with Tm or DMSO (Ctrl) before the experiments. Apoptosis was assessed as the percentage of apoptotic/total cell numbers. 8–20 pictures per treatment were randomly taken for analysis. 100-300 cells per picture were analyzed. N = 26–43 pictures, *p < 0.05, **p < 0.01, ***p < 0.001. (**D**) HEK293T cells were transfected with PC1-5TMC or GFP vector and 24 hours later transfected with GADD34 or empty vector (Ctrl). After 24 hours, TUNEL assays were similarly performed as in panel B. Statistical data were from the TUNEL assays and averaged to compare the apoptotic levels in the above treated HEK cells. Apoptosis indicates the percentage of apoptotic/total cell numbers. N = 26–41, **p < 0.01. (**E**) Representative data obtained from the same conditions as in panel D and showing the effects of GADD34 and PC1-5TMC on P-eIF2α.

Our WB data showed that PC1-5TMC suppresses P-PKR and P-eIF2α, with or without Tm treatment, while Tm increases the P-eIF2α level (Fig. 5A). TUNEL assays revealed that PC1-5TMC significantly represses apoptosis in the absence (P = 0.003) and presence of Tm treatment (P < 0.001) (Fig. 5B and C). Tm significantly increased the percentage of apoptotic cells (P < 0.001) (Fig. 5C), consistent with previous reports that P-eIF2α induces apoptosis^20, 21, 24^. To provide further documentations, we over-expressed growth arrest and DNA damage-inducible protein 34 (GADD34), a negative regulator of P-eIF2α, in HEK293T cells to promote lower level of P-eIF2α by protein phosphatase 1 (PP1). Indeed, while eIF2α phosphorylation and apoptosis were both repressed by GADD34, the inhibitory effect of PC1-5TMC on apoptosis was also abolished (Fig. 5D and E). Thus, these data together strongly indicated that a P-eIF2α pathway mediates the inhibition of cell apoptosis by PC1-5TMC.

**Figure 6:**
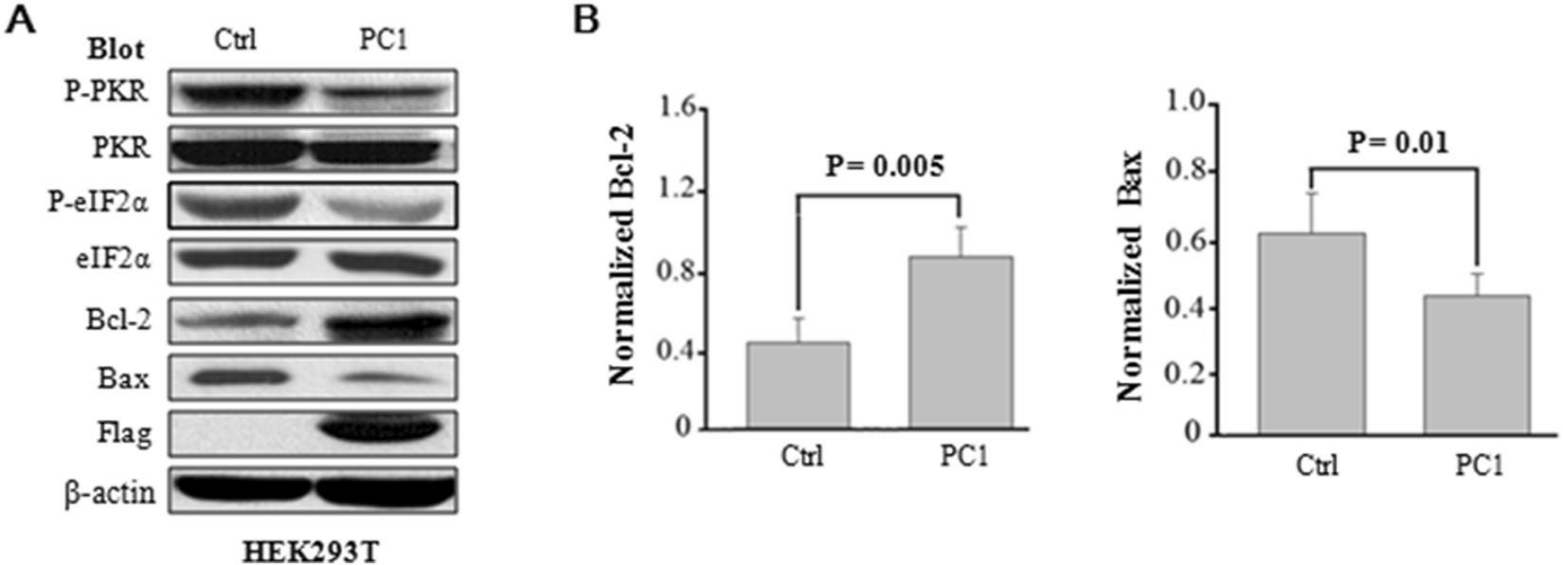
Effects of PC1 on expression of Bcl-2 and Bax. (**A**) Expression of different proteins in control and PC1 stable HEK293T cells after 48 hours of culture revealed by WB using different antibodies as indicated. β-actin served as loading controls. (**B**) *Left panel*, statistical data showing an averaged Bcl-2 level after normalization by β-actin (N = 5, P = 0.005, paired t-test). *Right panel*, data showing an averaged BAX level after normalization by β-actin (N = 5, P = 0.01, paired t-test).

We next sought to identify proteins implicated in the anti-apoptotic function of PC1. PKR were previously shown to activate Bax and inactivate Bcl-2^28–31^. The inhibitory effect of WT PC1 on cell apoptosis was also supported by our WB finding in HEK293T cells that it enhances the level of Bcl-2 while reducing the level of Bcl-2-like protein-4, Bax, presumably through inhibiting the PKR and eIF2α activities (Fig. 6A), which are in line with previous studies^28–31^. In average, WT PC1 increased the level of Bcl-2 by 43.9% ± 10.6% (p = 0.005, N = 5) and reduced that of Bax by 30.4% ± 12.5% (p = 0.01, N = 5) (Fig. 6B). These results are in support of the assumption that PC1 inhibits apoptosis through down-regulating P-PKR/P-eIF2α.

## Discussion

In this study we have shown that PC1 promotes lower levels of P-PKR and P-eIF2α using cultured cell lines and Pkd1−/− mouse embryonic kidney cells. In the presence of dominant negative PKR mutant K296R that loses the kinase activity, PC1 and PC1-5TMC did no longer exhibit any inhibitory effect on eIF2α phosphorylation, strongly indicating that PC1 down-regulates P-eIF2α through repressing the PKR activity. Previous studies have shown that PC1 is involved in regulation of de-phosphorylation through forming a holoenzyme complex with PP1 alpha (PP1α) via a conserved PP1α-binding motif within the polycystin-1 C-terminus, through it regulates downstream signaling^39^. For example, PC1 promotes lower level of PC2 at Ser829 through recruitment of PP1α^40^. It was also known that GADD34 functions as a scaffold to reduce the level of P-eIF2α by interacting with both PP1α and eIF2α^41^.

While we found in this study that PC1 and PC1-5TMC down-regulate P-eIF2α through repressing PKR kinase activity, our previous study found that PC2 promotes eIF2α phosphorylation through enhancing the efficiency of PERK as a kinase in phosphorylating eIF2α^33^. On the other hand, besides the involvement of PC1 in regulating apoptosis^8, 9^, both PC1 and PC2 are known to be anti-proliferative^33, 42^. Thus, the inhibition of cell proliferation by PC1 should be through a pathway independent of the PKR-eIF2α axis because regulation through this axis would lead to increased proliferation. Future experiments will explore whether and how PC1 regulates proliferation through a PKR-mTOR axis.

We noticed that in the presence of PKR knockdown by siRNA or over-expression of dominant negative mutant K296R PC1 does not further reduce the P-eIF2α level (Fig. 4C). We think that this is because under this condition the P-eIF2α level must be defined by other eIF2α kinases and phosphatases and thus be insensitive to PC1.

PC1 is known to induce resistance to apoptosis through PI3K/Akt and Gα_12_/JNK pathways^9, 10^ while the catalytic activity of PKR is known to promote phosphorylation of the PI3K pathway components such as Akt/protein kinase B (PKB), ribosomal protein S6 and eukaryotic initiation factor 4E binding protein 1 (4E-BP1). Reversely, induction of PI3K signaling antagonizes the pro-apoptotic and anti-synthesis effects of conditionally activated PKR^43^. Further, NF-κB as a downstream effector of PI3K/Akt was also shown to be involved in PC1-regulated apoptosis. For example, NF-κB inhibitor parthenolide reduced the anti-apoptotic effect of the PC1 cytoplasmic terminus^44^. On the other hand, it was found that PKR chronologically activates cell survival through NF-κB and cell death through eIF2α phosphorylation^45^. Therefore, how the PKR-eIF2α pathway is engaged in cross-talk with the PI3K/Akt and other signaling pathways with respect to regulation of cell apoptosis by PC1 is subject to future studies. In summary, our data were in support that PC1 inhibits cell apoptosis through a pathway that depends on the P-PKR/P-eIF2α axis and its downstream factors, anti-apoptotic Bcl-2 and pro-apoptotic BAX. Further studies should examine which apoptosis pathway downstream of P-eIF2α that is implicated in mediating the anti-apoptotic effect of PC1.

## Materials and Methods

### Antibodies and reagents

Rabbit antibodies against Phospho-eIF2α (Ser51), eIF2α, PKR were purchased from Cell Signaling Technology (New England Biolabs, Pickering, ON). Phospho-PKR (pT446), P-PKR (Thr 446), PKR (B-10), PACT, Bcl-2, Bax antibodies were from Epitomics (Burlingame, CA) or Santa Cruz (Santa Cruz, CA). Affinity purified goat polyclonal anti-GFP (EU4) (Eusera, Edmonton, AB) was utilized for immunoprecipitation and mouse monoclonal anti-GFP (B-2) (Santa Cruz) for immunoblotting using GFP-tagged plasmids. Rabbit (A2066, Sigma-Aldrich Canada, Oakville, ON) or mouse anti-β-actin (C4, Santa Cruz), goat anti-FLAG (Santa Cruz) and mouse monoclonal anti-HSP60 (H-1) (Santa Cruz) antibodies were used for loading controls. Secondary antibodies were purchased from GE Healthcare (Baie d’Urfe, Quebec) or Santa Cruz. Tm and IFN-α were from Sigma-Aldrich Canada.

### Cell culture, DNA constructs and transfection

HEK293T and HeLa cells were cultured in Dulbecco’s modified Eagle’s medium (DMEM) supplemented with L-glutamine, penicillin-streptomycin, and 10% fetal bovine serum at 37 °C and 5% CO_2_. HEK293T cells stably expressing Flag-tagged full length mouse PC1 was a generous gift of Dr. J. Yang (Columbia University, NY) and cultured under above-described medium supplemented with 2 µg/ml of puromycin (Sigma-Aldrich Canada)^46^. pcDNA3 plasmids encoding the N-terminal GFP-tagged PC1 C-terminus (PC1C, aa 4104-4302), last 5 TMs plus PC1C (PC1-5TMC, aa 3895–4302), and last TM plus PC1C (PC1-1TMC, aa 4084–4302) were constructed by site-directed mutagenesis using Stratagene QuikChange® II XL Site-Directed Mutagenesis Kit (Agilent Technologies Canada Inc., Mississauga, ON). HEK293T cells were grown to ~70% confluency prior to transfection using Lipofectamine 2000 (Invitrogen Canada Inc., Burlington, ON). All plasmid construction and cDNA sequences were verified by sequencing.

### Co-IP

Protein extraction, immunoblotting and co-IP were performed as described previously^33, 47, 48^. Typically, 20 and 200 mg of total cellular protein were used for immunoblotting and co-IP, respectively. HEK293T or HeLa cells were transiently transfected with pEGFP or pEGFP-PC1C for co-IP assays. At 40 hours post-transfection, cells were used for protein extraction and precipitation.

### Cell surface biotinylation

Cell surface biotinylation was performed as previously with modifications^46, 47^. Briefly, HeLa cells transfected with GFP vector, GFP-PC1-5TMC, or GFP-PC1-1TMC, were grown to 90% confluency in 60 mm dishes. Cells were washed with ice-cold PBS then borate buffer (10 mM boric acid, 154 mM NaCl, 7.2 mM KCl, 1.8 mM CaCl_2_, pH 9.0), and incubated with 1 mg/ml Pierce EZ-Link^TM^ Sulfo-NHS-SS-Biotin (Fisher Scientific Canada, Toronto, ON) at 4 °C with agitation for 30 minutes. After washing with quenching buffer (192 mM glycine, 25 mM Tris, pH 8.3), cells were lysed in ice-cold CelLytic^TM^-M reagent supplemented with protease inhibitor cocktail (Sigma-Aldrich Canada). The biotinylated and flow-through intracellular proteins were separated using 100 µl of Pierce Avidin Agarose (Fisher Scientific Canada) by incubation overnight at 4 °C and subsequent centrifugation. After intensive washing in NP40 buffer (50 mM Tris pH 7.5, 150 mM NaCl, 1% NP40) with protease inhibitor cocktail, biotinylated proteins were resuspended in 5 x SDS sample buffer and eluted from beads by heating at 65 °C for 5 minutes. An equal amount of biotinylated proteins and 20 µg of total and intracellular proteins were separated on 8% sodium dodecyl sulfate-polyacrylamide gel electrophoresis (SDS-PAGE) for immunoblotting.

### Gene knockdown by siRNA

PKR siRNA (Santa Cruz, Cat#sc-36263) was used to transfect HEK293T cells using Lipofectamine 2000 reagent following the manufacturer’s instructions. The efficiency of the siRNA knockdown was assessed by immunoblotting.

### TUNEL assay

HEK293T cells were transfected with GFP or GFP-PC1-5TMC in 100 mm dishes. At 24 hours post-transfection, cells were split and grown on coverslips and subject to 2.5 µM of Tm treatment or GADD34 transfection for 24 hours. Then cells were fixed with 2% paraformaldehyde for 10 minutes at room temperature and washed twice with PBS. Cells were then permeated with PBS containing 0.05% Triton X-100 for 3 minutes at room temperature, and subject to TUNEL assay using DeadEnd™ Colorimetric TUNEL System according to the manufacturer’s instructions (Promega North America, Madison, WI).

### Data analysis

WB bands were quantified by Image J (National Institute of Health, Bethesda, MD) and data were expressed as mean ± SEM (N), analyzed and plotted using SigmaPlot 12 **(**Systat Software Inc., San Jose, CA), where SEM represents the standard error of the mean and N indicates the number of experimental repeats. A probability value (P) of less than 0.05 and 0.01 was considered significant (*) and very significant (**), respectively.
